# Spatio-temporal variations of extract produced and fatty acid compounds identified of *Gundelia tournefortii* L. seeds in central Zagros, Iran

**DOI:** 10.1038/s41598-023-34538-5

**Published:** 2023-05-11

**Authors:** H. R. Karimzadeh, H. R. Farhang, M. Rahimmalek, M. Tarkesh Esfahani

**Affiliations:** 1 Department of Natural Resources, Isfahan University of Technology, Isfahan, 84156–83111 Iran; 2 Department of Horticulture, College of Agriculture, Isfahan University of Technology, Isfahan, 84156–83111 Iran

**Keywords:** Biogeochemistry, Lipids, Biochemistry, Environmental sciences, Ecology, Biogeography, Ecosystem ecology

## Abstract

This study was performed to fulfill two aims. The first aim was to isolate the seed extract of *Gundelia tournefortii* L. at two phenological stages of seed production (the beginning and end of seed production); the second one was to identify the fatty acid compounds of *G. tournefortii* L. seeds in its major habitats located in the Central Zagros region, Iran. Among them, some of the major environmental factors on the reproductive growth stage i.e., physiography, soil and climate were studied. Extraction was performed using the Soxhlet apparatus, and the fatty acid compounds were identified by The GC-FID analysis. As a result, site No. 5 with the values of 6.06 and 7.21 g had the highest amount of extract produced, while sites number 7 and 8 had the least one which was 2.86 and 3.84 g at two phenological stages of seed production. There was a strong correlation among the major environmental variables and the amount of extract produced in the phenological stages of seed production; this was also confirmed in relation to the fatty acid compounds and some of their characteristics. Overall, the efficacy of environmental factors on the synthesis process of secondary metabolites is undeniable.

## Introduction

At the end of the nineteenth century, due to the increasing advances in different sciences, especially chemistry, coupled with its extensive field and pharmaceuticals, the first extraction of pure chemical materials for medicinal utilization was introduced which led to the treatment of patients, miraculously^1^. Following that, the tendency for medicinal plants’ consumption increased in recent decades considerably, so the twenty-first century could be named the studying and consuming medicinal plants era^2^.

Secondary metabolites of the medicinal plants are processed subject to genetic processes' original control, while the production of the mentioned compounds is affected by environmental factors. It is believed that secondary metabolites are produced to regulate plant adaptations against unfavorable factors and environmental stresses, and are extracted for the chemical defense to keep balance and continue the plant’s vital activities^3^.

Fats and all types originated from plants and animals are the most important components of the nutrient sources^4^. The fatty acids consist of: (1) saturated, (2) unsaturated single-bond, and (3) unsaturated multi-bonds. Fatty acids are categorized based on the length of the chain, the double bonds count, or its unsaturated degree in the chain^5^. Among these, the omega-3, omega-6, and omega-9 fatty acids compositions belong to the two major classes of the mentioned compounds, namely polyunsaturated fatty acids (PUFAs) and monounsaturated fatty acids (MUFAs)^6^. The human body needs these essential fatty acids (EFAs) for its biological processes. The omega-3 fatty acids are beneficial for heart, brain, and metabolism activities. The omega-6 fatty acids are an important source of energy for the human body and the omega-9 fatty acids must exist to a lower extent because they are produced by the human body to promote metabolic health^7^.

Most biochemists and ecologists have expanded their research in the field of identification and analysis of different ecological factors in association with the quantity and quality of secondary metabolites and natural bioactive compounds^8^. The ecological factors might affect the proprietary enzymes related to the biochemical synthesis pathway of secondary metabolites and reduce the contribution stability^9^. In this context, the most important ecological factors on the quantity and quality of the secondary metabolites and natural bioactive compounds are categorized as climatic, edaphic, and physiographic. Each of these factors is made of different components, creating different environmental gradients in the study of the subject sites ecologically^10^. The genetic and inheritance factors like the diversity of the inter- and intra- plant species, the variety among cultivars, different genotypes, and the adoption of the methods and techniques of crop improvement and breed improvement contribute highly to the production rate of secondary metabolites^11^. The integration of the genetic and environmental factors in ecological studies can provide favorable results in achieving a comprehensive analysis of the quantity and quality of secondary metabolites and natural bioactive compounds^12^.

Iran due to being located in a special geographical region is subject to the emergence of different climates and edaphic conditions with a unique place in the plant geography in terms of plant diversity at global status. Iran is one of the 10 most important origins-specific plants. Moreover, there is a field of growing diverse plants with different ecological characteristics like edible, medicinal, and industrial plants in Iran with a recorded history of thousands of years^13^.

The Asteraceae is the largest family of flowering plants with about 900 genera and more than 13,000 species. Genus *Gundelia*, from Asteraceae, has only one species in Iran, named the *Gundelia tournefortii* L^14^. The genus *Gundelia* is a perennial, vigorous, and succulent plant with alternate leaves and pinnate divisions by serrated sides which are converted into thorns. The upper leaves surround and cover the stems of the capitols, which have tubular flowers of the monoecious, placed next to each other in the form of a spherical collection. Each capitol has a major bract with a collar consisting of many rows of leaflets connected, which create an inverted conical bowl with thorny edges. The receptacle is covered by a connected scale-like straw forming cells, cavities, and chambers by the collar bracts, where a flower is placed inside. The flowers come in green, yellow, white, pink, and purple. The capitols eventually become woody and indehiscent^14^. This plant grows in the mountainous, tropical, or temperate region. The highest distribution of this plant on the global scale is reported in the countries around the Mediterranean Sea, African countries, the Middle East, Afghanistan, Turkmenistan, and beyond Caucasus^14^. It is emphasized that there is no evidence of consuming the secondary metabolites of *G. tournefortii* L. to make a formulated herbal drug registered in the Iranian Pharmacopoeia. It is also accentuated that no license has been issued by the Iranian Ministry of Health, Medicine and Medical Education regarding the medicinal products or the synthesized herbal drug obtained from the secondary metabolites of *G. tournefortii* L. for general use.

The objective of this study is to identify the fatty acid profiles of *G. tournefortii* L. seeds and their features through the soxhlet apparatus and assess them by GC/FID. The assessment and analyses would allow the understanding of some of the most important ecological variables (climate, soil, and physiography) on the volume of produced extract through the PCA and cluster analysis among the major habitats of *G. tournefortti* L. located in 11 major habitats of the Central Zagros region of Iran. The identified fatty acid compounds of *G. tournefortii* L. seeds and their features were assessed and analyzed through the aforementioned techniques.

## Results

### Extraction of *G. tournefortii* L. seed oil during phenological stages

In general, extraction of *G. tournefortii* L. seed oil is run out in two different phonological stages of reproductive growth, namely the beginning of seed production and the end of seed production for the subject habitats. The colors of the oily extract in each habitat vary in the yellow spectrum. The results indicate that the yield of extract volume in these two mentioned phenological stages are different in their quantitative sense, something that does not hold for the qualitative state. As to the quantitative volume of the obtained extract at the beginning of seed production stages (5.72 g) at site No. 4 and (6.06 g) at site No. 5 with an average of (7.21 g) is evident; while as to the quantitative volume of the obtained extract at the end of seed production stages at the sites No. 4 and No. 5 the averages are (6.88 g) and (7.21 g), respectively. It could be stated that the mentioned sites are superior in terms of extract volume. As to the lowest quantitative volume, sites are at both the beginning and the end of seed production stages. The lowest quantitative volume is recorded from sites No. 7 with an average of (2.86 g) and No. 11 (2.92 g) at the beginning of the seed production stage. Meanwhile, the lowest quantitative amount of the extract produced by *G. tournefortii* L. seeds was reported from sites No. 6, with an average (of 3.84) and No. 11 (3.86) at the end of the seed production stage. Among the study sites, sites No. 7 and No. 11 at the beginning of the seed production stage and sites No. 6 and No. 11 at the end of the seed production stage had the least amount of extract produced of *G. tournefortii* L. seeds (Table 1).

**Table 1:** Mean comparisons of the amount of extract produced from *G. tournefortii* L. seeds based on two phenological stages of seed production in the study sites.

Samples	The amount of extract produced from *G. tournefortii* L. seeds at the beginning of seed production (g)	The amount of extract produced from *G. tournefortii* L. seeds at the end of seed production (g)
Site 1	4.13 ± 0.04^e^	5.24 ± 0.06^d^
Site 2	3.21 ± 0.04^c^	4.17 ± 0.06^b^
Site 3	3.95 ± 0.07^d^	5.37 ± 0.04^d^
Site 4	5.72 ± 0.08^ g^	6.88 ± 0.05^e^
Site 5	6.06 ± 0.08^ h^	7.21 ± 0.05f.
Site 6	4.33 ± 0.06f.	3.84 ± 0.06^a^
Site 7	2.86 ± 0.06^a^	4.16 ± 0.06^b^
Site 8	3.20 ± 0.04^c^	4.12 ± 0.06^b^
Site 9	3.00 ± 0.08^b^	5.30 ± 0.05^d^
Site 10	4.17 ± 0.06^e^	4.83 ± 0.04^c^
Site 11	2.92 ± 0.09^a^	3.86 ± 0.10^a^

Mean ± standard deviation (n = 3). Tukey's test was performed to compare the means (*p* < 0.05).

The results obtained from comparing the means among the study sites revealed no significant difference in sites No. 7, 11, 1, and 10 in terms of the yield extract at the beginning of the seed production stage; while in the remaining sites Nos.: that the other seven sites namely 2, 3, 4, 5, 6, 8 and 9 had a significant difference from each other. The study sites, 6 and 11, 2, 7 and 8, and 1, 3, and 9 are not significantly different in terms of the extract volume at the end of the seed production stage. The results indicate that sites 4, 5, and 10 had a significant difference. Overall, comparing the trend of variations in terms of the yield extract volume in these process reveals that the found percentage at the end of the seed production stage are higher than the beginning stage. The mentioned increasing trend is these percentages alter within (7.33%) in site No. 10 to (27.71%) in No. 9. It is observed that only at site No. 6, the extracted content at the end of the seed production stage at (5.99%) was less than all other sites (Table 2).

**Table 2:** Comparison of alterations of the extract percentage in two phenological stages of seed production of *G. tournefortii* L. in the study sites.

Samples	The extract percentage of *G. tournefortii* L. seeds at the beginning of seed production	Percentage of the amount of extract produced from *G. tournefortii* L. seeds at the end of seed production	The difference between the calculated percentages of the amount of extract produced from *G. tournefortii* L. seeds
Site 1	44.07	55.92	11.85
Site 2	43.49	56.50	13.01
Site 3	42.38	57.61	15.23
Site 4	45.39	54.60	9.21
Site 5	45.66	54.33	8.67
Site 6	52.99	47	5.99
Site 7	40.74	59.25	18.51
Site 8	43.71	56.28	12.57
Site 9	36.14	63.85	27.71
Site 10	46.33	53.66	7.33
Site 11	43.06	56.93	13.87

### The identified fatty acids compounds of *G. tournefortii* L. seeds and their features in the same duration

In this study, the following six fatty acid compounds are identified: myristic acid (C14:0; tetradecanoic acid), palmitic acid (C16:0; hexadecanoic acid), stearic acid (C18:0; octadecanoic acid), oleic acid (C18:1; 9-octadecenoic acid), linoleic acid (C18:2; 9,12–octadecadienoic acid) and linolenic acid (C18:3; 9,12,15–octadecatrienoic acid). These identified fatty acid compounds are matched vs. the available reference samples. All experiments were performed for both phenological stages of seed production of *G. tournefortii* L. in the study sites, separately (Supplementary Fig. 1 and Supplementary Fig. 2).

In general, the identified fatty acids are not significantly different in their qualitative sense in both phenological stages, while in the quantitative sense, they are identified at both phenological stages. These compounds are different from each other, in their features and quantitative sense. Among these compounds, the two linoleic acids as the highest fatty acid compounds and meristic acid as the lowest, are identified and recorded at both phenological stages.

The highest volume of these fatty acids is attributed to oleic acid, palmitic acid, and stearic acids. These compounds have the same general superiority in both phenological stages of seed production of *G. tournefortii* L. The two remaining compounds in the fatty acid contents, linoleic acid, and myristic acid, are recorded as having the lowest content in both phenological stages. The quantitative volume obtained from these two is different from each other in both phenological stages. The differences in the quantitative values of the identified fatty acid profiles are generally attributed to two main factors, the genetic characteristics of the plant and the ecological properties primary factor in the study sites.

The secondary metabolites, constituents, and by-products of medicinal plants are originally produced subject to the control of genetic processes influenced by environmental factors. Because their contribution to plants is not clear; it is believed that secondary metabolites are primarily produced to regulate the plant’s adaptation to adverse factors and environmental tensions^18^. The environmental factors cause changes in the synthesis procedure and production of various constituents of medicinal plants, both quantitatively and qualitatively^18^. The cultivation of medicinal plants is considered cost-effective when the production of primary and secondary metabolites reported in the plant is at its optimum^17^.

Some of the most essential features of these fatty acids are: Saturated fatty acids (SFAs), unsaturated fatty acids (UFAs), mono-bonded unsaturated fatty acids (MUFAs), and poly-bonded unsaturated fatty acids groups (PUFAs), the linoleic acid to linolenic acid (n-6/n-3) ratio, the Unsaturated fatty acids to saturated fatty acids (UFAs/SFAs) ratio, the polyunsaturated fatty acids to saturated fatty acids (PUFAs/SFAs) ratio, the monounsaturated fatty acids to polyunsaturated fatty acids (MUFAs/PUFAs) ratio and the Cox value index “Eq. (1)” in both two phenological stages of seed production of *G. tournefortii* L. are studied and analyzed in all of the sites. The cox value index is calculated in the percent of 18-carbon unsaturated fatty acids^19^.

1 $$ {\text{Cox Value}} = \frac{{\left[ {1\left( {{\text{C}}18:1{\text{\% }}} \right){ } + { }10.3\left( {{\text{C}}18:2{\text{\% }}} \right){ } + { }21.6\left( {{\text{C}}18:3{\text{\% }}} \right)} \right]}}{100} $$

In the above equation, C18:1, C18:2 and C18:3 are oleic, linoleic and linolenic fatty acids, respectively.

The results of the mean comparison among the study sites, together with their relevant details are tabulated in Table 3 (the beginning of the seed production stage) and Table 4 (the end of the seed production stage). As to the findings regarding the beginning stage, the following identified fatty acid types are tabulated in Table 3: myristic acid, palmitic acid, and stearic acid were identified as the three saturated fatty acids. The highest and the lowest volumes of myristic acid are reported in the study sites No. 9 (0.57%) and No. 1 (0.001%). The highest content of palmitic acid is reported in sites No. 9 (14.48%), No. 11 (11.90%), and No. 4 (10.80%), respectively. The lowest content of the mentioned compounds is recorded in site No. 1 at (9.76%). The highest volume of stearic acid is observed in site No. 11 at (3.69%), and the lowest is reported in site No. 9 at (1.88%). The highest volumes of SFAs are reported from site No. 9 at (16.94%), site No. 11 at (15.63%) and site No. 4 at (13.96%). The lowest content of SFAs is attributed to site No. 1 at (12.66%). The highest volume of MUFAs is recorded for sites No. 11 at (40.2%), No. 4 at (38.28%) and No. 3 at (38.09%). The lowest volume is observed in site No. 9 at (31.7%). As to the PUFAs, the highest volume is attributed to linoleic acid and the lowest to linolenic acid, sites No. 6 at (51.51%), No. 1 at (51.16%) and No. 5 at (51.07%) contain the highest volumes of linoleic acid. The lowest volume is recorded for site No. 4 at (47.6%). The highest volume of linolenic acid is obtained from site No. 9 at (2.53%). The lowest volume is obtained from the sites No. 5 and 8 both at (0.09%). It is revealed that, the highest volume of PUFAs is attributed to site No. 1 at (52.28%), site No. 6 at (51.60%) and site No. 9 at (51.32%). The lowest volume is attributed to site No. 11 at (44.25%).The results of UFAs reveale that the highest volume of the mentioned compounds is attributed to sites No. 1 at (87.32%), No. 6 at (87.29%), No. 10 at (87.04%) and No. 5 at (87.02%). It is while that the lowest amount of the mentioned compounds was reported from site No. 9 (83.05%).The linoleic acid to linolenic acid (n-6/n-3) ratio in all sites are significantly different from each other as the highest volumes are attributed to site No. 8 at (722.87) and the lowest to site No. 9 at (19.45). The UFAs to SFAs ratio reveal that the highest volumes are attributed to site No.1 at (6.89) and site No. 6 at (6.88).The lowest volume is attributed to reported from site No. 9 at (4.9). The PUFAs to SFAs ratio indicate that site No. 1 at (4.12) and site No. 6 at (4.07) are the highest. The lowest ratio is attributed to site No. 11 at (2.83). The MUFAs to PUFAs ratio indicates that the highest volume is of site No. 11 at (0.9) and the lowest is of site No. 9 at (0.61). The results of Cox value index indicates that site No. 9 at (5.85) has the highest volume among all sites. The lowest volume of the mentioned index is attributed to site No. 11 at (5.01). The Cox value index of the other sites flactuate between the lowest and the highest volumes at the beginning of seed production stage.

**Table 3:** Fatty acids profile and their features (%) obtained from GC-FID analysis of *G. tournefortii* L. seeds at the beginning of seed production stage in the study sites.

Samples	Site 1	Site 2	Site 3	Site 4	Site 5	Site 6	Site 7	Site 8	Site 9	Site 10	Site 11
Fatty acids	Saturated fatty acids (SFA)										
C14:0	0.001 ± 0.0004^a^	0.01 ± 0.004^a^	0.08 ± 0.10^a^	0.02 ± 0.002^a^	0.003 ± 0.001^a^	0.08 ± 0.02^a^	0.02 ± 0.003^a^	0.07 ± 0.03^a^	0.57 ± 0.03^b^	0.002 ± 0.001^a^	0.04 ± 0.01^a^
C16:0	9.76 ± 0.05^a^	10.24 ± 0.05^d^	10.14 ± 0.03^d^	10.80 ± 0.02^g^	10.29 ± 0.03^e^	10.05 ± 0.04^c^	10.56 ± 0.02^f^	10.49 ± 0.02^f^	14.48 ± 0.05^i^	9.89 ± 0.02^b^	11.90 ± 0.05^h^
C18:0	2.90 ± 0.03^d^	3.03 ± 0.03^e^	3.05 ± 0.02^f^	3.13 ± 0.02^f^	2.67 ± 0.01^c^	2.55 ± 0.04^b^	2.71 ± 0.02^c^	2.73 ± 0.02^c^	1.88 ± 0.05^a^	3.05 ± 0.02^f^	3.69 ± 0.02^g^
Total SFA	12.66 ± 0.03^a^	13.28 ± 0.02^c^	13.21 ± 0.02^c^	13.96 ± 0.03^d^	12.96 ± 0.03^b^	12.68 ± 0.02^a^	13.29 ± 0.03^c^	13.29 ± 0.02^c^	16.94 ± 0.03^f^	12.94 ± 0.04^b^	15.63 ± 0.04^e^
	Monounsaturated fatty acids (MUFA)										
C18:1	36.02 ± 0.25^d^	36.63 ± 0.02^e^	38.09 ± 0.02^f^	38.28 ± 0.07^f^	35.85 ± 0.03^c^	35.63 ± 0.03^b^	36.19 ± 0.02^d^	36.02 ± 0.02^d^	31.7 ± 0.06^a^	36.72 ± 0.03^e^	40.2 ± 0.03^g^
Total MUFA	36.02 ± 0.25^d^	36.63 ± 0.02^e^	38.09 ± 0.02^f^	38.28 ± 0.07^f^	35.85 ± 0.03^c^	35.63 ± 0.03^b^	36.19 ± 0.02^d^	36.02 ± 0.02^d^	31.7 ± 0.06^a^	36.72 ± 0.03^e^	40.2 ± 0.03^g^
	Polyunsaturated fatty acids (PUFA)										
C18:2	51.16 ± 0.22^h^	49.85 ± 0.03^e^	48.51 ± 0.01^c^	47.6 ± .02^b^	51.07 ± 0.02^h^	51.51 ± 0.03^i^	50.39 ± 0.03^g^	50.57 ± 0.03^g^	48.52 ± 0.04^d^	50.09 ± 0.04^f^	48.32 ± 0.03^a^
C18:3	0.14 ± 0.02^b^	0.21 ± 0.02^c^	0.16 ± 0.03^c^	0.12 ± 0.02^a^	0.09 ± 0.02^a^	0.15 ± 0.03^b^	0.11 ± 0.02^a^	0.09 ± 0.04^a^	2.53 ± 0.03^e^	0.22 ± 0.03^c^	0.33 ± 0.02^d^
Total PUFA	52.28 ± 0.84^ h^	50.01 ± 0.04^d^	48.58 ± 0.10^c^	47.77 ± 0.05^b^	51.16 ± 0.03^g^	51.60 ± 0.03^g^	50.49 ± 0.03^e^	50.69 ± 0.02^f^	51.32 ± 0.02^g^	50.31 ± 0.03^d^	44.25 ± 0.03^a^
	Total unsaturated fatty acids (UFA)										
Total UFA	87.32 ± 0.04^f^	86.73 ± 0.07^d^	86.77 ± 0.02^d^	86.02 ± 0.03^c^	87.02 ± 0.03^e^	87.29 ± 0.02^f^	86.69 ± 0.03^d^	86.69 ± 0.02^d^	83.05 ± 0.03^a^	87.04 ± 0.05^e^	84.35 ± 0.04^b^
n-6/n-3 ratio*	366.71 ± 0.06^h^	207.6 ± 0.02^c^	303.26 ± 0.04^e^	317.57 ± 0.01^f^	468.18 ± 0.03^j^	343.2 ± 0.03^g^	458.1 ± 0.02^i^	722.87 ± 0.02^k^	19.45 ± 0.03^a^	227.75 ± 0.08^d^	121.84 ± 0.01^b^
UFA/SFA ratio	6.89 ± 0.02^f^	6.52 ± 0.01^d^	6.55 ± 0.01^d^	6.15 ± 0.01^c^	6.7 ± 0.02^e^	6.88 ± 0.01^f^	6.51 ± 0.02^d^	6.51 ± 0.01^d^	4.9 ± 0.01^a^	6.72 ± 0.03^e^	5.38 ± 0.01^b^
PUFA/SFA ratio	4.12 ± 0.07^h^	3.76 ± 0.01^e^	3.67 ± 0.01^d^	3.42 ± 0.01^c^	3.95 ± 0.01^g^	4.07 ± 0.01^h^	3.79 ± 0.005^e^	3.81 ± 0.01^f^	3.03 ± 0.02^b^	3.88 ± 0.02^f^	2.83 ± 0.01^a^
MUFA/PUFA ratio	0.66 ± 0.02^b^	0.73 ± 0.00^d^	0.78 ± 0.005^e^	0.79 ± 0.005^e^	0.69 ± 0.005^c^	0.69 ± 0.00^c^	0.71 ± 0.005^d^	0.7 ± 0.005^d^	0.61 ± 0.005^a^	0.72 ± 0.005^d^	0.9 ± 0.005^f^
Cox value index	5.72 ± 0.06^g^	5.52 ± 0.01^d^	5.4 ± 0.02^c^	5.3 ± 0.02^b^	5.62 ± 0.01^f^	5.67 ± 0.02^g^	5.57 ± 0.02^e^	5.60 ± 0.02^f^	5.85 ± 0.03^h^	5.57 ± 0.03^e^	5.01 ± 0.02^a^

*The ratio of omega-6 to omega-3 fatty acids group. Mean ± standard deviation (n = 3). Tukey's test was performed to compare the means (*p* < 0.05).

**Table 4:** Fatty acids profile and their features (%) obtained from GC-FID analysis of *G. tournefortii* L. seeds at the end of seed production stage in the study sites.

Samples	Site 1	Site 2	Site 3	Site 4	Site 5	Site 6	Site 7	Site 8	Site 9	Site 10	Site 11
Fatty acids	Saturated fatty acids (SFA)										
C14:0	0.01 ± 0.003^a^	0.10 ± 0.03^c^	0.07 ± 0.05^c^	0.03 ± 0.01^b^	0.03 ± 0.002^b^	0.03 ± 0.002^b^	0.01 ± 0.002^a^	0.03 ± 0.004^b^	0.004 ± 0.002^a^	0.002 ± 0.001^a^	0.04 ± 0.005^b^
C16:0	10.15 ± 0.07^b^	10.81 ± 0.02^d^	10.37 ± 0.02^c^	12.01 ± 0.03^g^	11.16 ± 0.05^e^	11.37 ± 0.06^f^	9.37 ± 0.02^a^	13.5 ± 0.01^i^	12.61 ± 0.03^h^	10.15 ± 0.06^b^	10.17 ± 0.01^b^
C18:0	2.58 ± 0.13^a^	2.93 ± 0.02^c^	2.72 ± 0.03^b^	3.75 ± 0.05^f^	3.26 ± 0.02^e^	3.13 ± 0.03^d^	2.76 ± 0.01^b^	3.79 ± 0.19^f^	3.61 ± 0.07^f^	2.95 ± 0.03^c^	2.99 ± 0.03^c^
Total SFA	12.78 ± 0.03^b^	13.85 ± 0.02^e^	13.16 ± 0.02^c^	15.80 ± 0.25^g^	14.46 ± 0.03^f^	14.54 ± 0.03^f^	12.15 ± 0.03^a^	17.45 ± 0.03^i^	16.23 ± 0.04^h^	13.11 ± 0.03^c^	13.21 ± 0.04^d^
	Monounsaturated fatty acids (MUFA)										
C18:1	35.86 ± 0.02^b^	38.36 ± 0.04^f^	35.89 ± 0.02^b^	30.75 ± 0.04^a^	38.33 ± 0.02^f^	39.32 ± 0.05^g^	36.09 ± 0.01^c^	40.9 ± 0.06^h^	41.19 ± 0.03^i^	36.94 ± 0.04^e^	36.32 ± 0.05^d^
Total MUFA	35.86 ± 0.02^b^	38.36 ± 0.04^f^	35.89 ± 0.02^b^	30.75 ± 0.04^a^	38.33 ± 0.02^f^	39.32 ± 0.05^g^	36.09 ± 0.01^c^	40.9 ± 0.06^h^	41.19 ± 0.03^i^	36.94 ± 0.04^e^	36.32 ± 0.05^d^
	Polyunsaturated fatty acids (PUFA)										
C18:2	51.23 ± 0.03^i^	47.55 ± 0.02^e^	50.53 ± 0.01^h^	53.22 ± .04^k^	46.98 ± 0.03^d^	45.92 ± 0.04^c^	51.63 ± 0.02^j^	41.33 ± 0.03^a^	42.08 ± 0.04^b^	49.88 ± 0.06^f^	50.39 ± 0.03^g^
C18:3	0.11 ± 0.02^a^	0.22 ± 0.03^b^	0.39 ± 0.02^d^	0.19 ± 0.02^b^	0.2 ± 0.02^b^	0.29 ± 0.02^c^	0.11 ± 0.02^a^	0.3 ± 0.02^c^	0.48 ± 0.03^e^	0.05 ± 0.01^a^	0.06 ± 0.02^a^
Total PUFA	52.12 ± 0.67^f^	47.77 ± 0.05^c^	50.97 ± 0.03^e^	53.47 ± 0.04^g^	47.19 ± 0.05^c^	46.21 ± 0.02^b^	51.68 ± 0.02^f^	41.61 ± 0.02^a^	42.16 ± 0.04^a^	49.89 ± 0.04^d^	50.47 ± 0.04^e^
	Total unsaturated fatty acids (UFA)										
Total UFA	87.21 ± 0.02^h^	86.13 ± 0.04^e^	86.81 ± 0.02^g^	84.17 ± 0.02^c^	85.52 ± 0.02^d^	85.44 ± 0.03^d^	87.83 ± 0.03^i^	82.53 ± 0.03^a^	83.76 ± 0.04^b^	86.88 ± 0.03^g^	86.77 ± 0.04^f^
n-6/n-3 ratio*	365.67 ± 0.04^h^	190.18 ± 0.19^e^	120.31 ± 0.04^b^	242.12 ± 0.04^g^	204.41 ± 0.03^f^	143.39 ± 0.02^d^	516.1 ± 0.04^i^	137.66 ± 0.03^c^	93.51 ± 0.03^a^	711.84 ± 0.04^j^	720 ± 0.02^k^
UFA/SFA ratio	6.82 ± 0.02^h^	6.21 ± 0.02^e^	6.59 ± 0.01^g^	5.33 ± 0.01^c^	5.91 ± 0.01^d^	5.87 ± 0.02^d^	7.22 ± 0.02^i^	4.72 ± 0.01^a^	5.15 ± 0.01^b^	6.62 ± 0.02^g^	6.56 ± 0.02^f^
PUFA/SFA ratio	4.07 ± 0.04^i^	3.44 ± 0.01^f^	3.87 ± 0.01^h^	3.38 ± 0.01^e^	3.26 ± 0.01^d^	3.17 ± 0.005^c^	4.25 ± 0.01^j^	2.38 ± 0.02^a^	2.60 ± 0.01^b^	3.8 ± 0.01^g^	3.81 ± 0.01^h^
MUFA/PUFA ratio	0.66 ± 0.02^b^	0.8 ± 0.00^f^	0.7 ± 0.00^d^	0.57 ± 0.00^a^	0.81 ± 0.005^f^	0.84 ± 0.005^g^	0.69 ± 0.005^c^	0.98 ± 0.00^h^	0.98 ± 0.005^h^	0.73 ± 0.005^e^	0.71 ± 0.005^e^
Cox value index	5.63 ± 0.02^g^	5.34 ± 0.02^e^	5.64 ± 0.02^g^	5.86 ± 0.02^h^	5.26 ± 0.01^d^	5.18 ± 0.02^c^	5.83 ± 0.03^h^	4.73 ± 0.02^a^	4.83 ± 0.03^b^	5.5 ± 0.02^f^	5.58 ± 0.02^g^

*The ratio of omega-6 to omega-3 fatty acids group. Mean ± standard deviation (n = 3). Tukey's test was performed to compare the means (*p* < 0.05).

**Figure 1:**
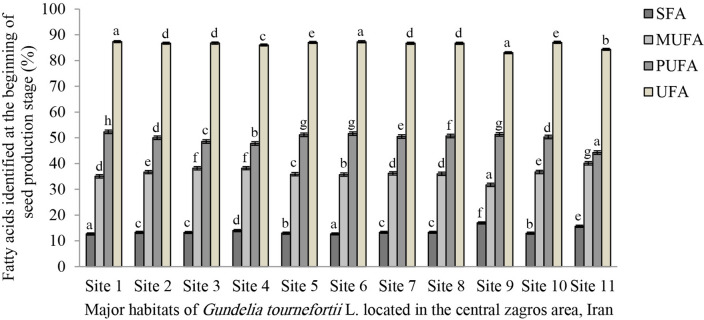
Bar chart of the quantitative amounts of SFA, MUFA, PUFA and UFA at the beginning of the seed production stage of *G. tournefortii* L. in the study sites.

**Figure 2:**
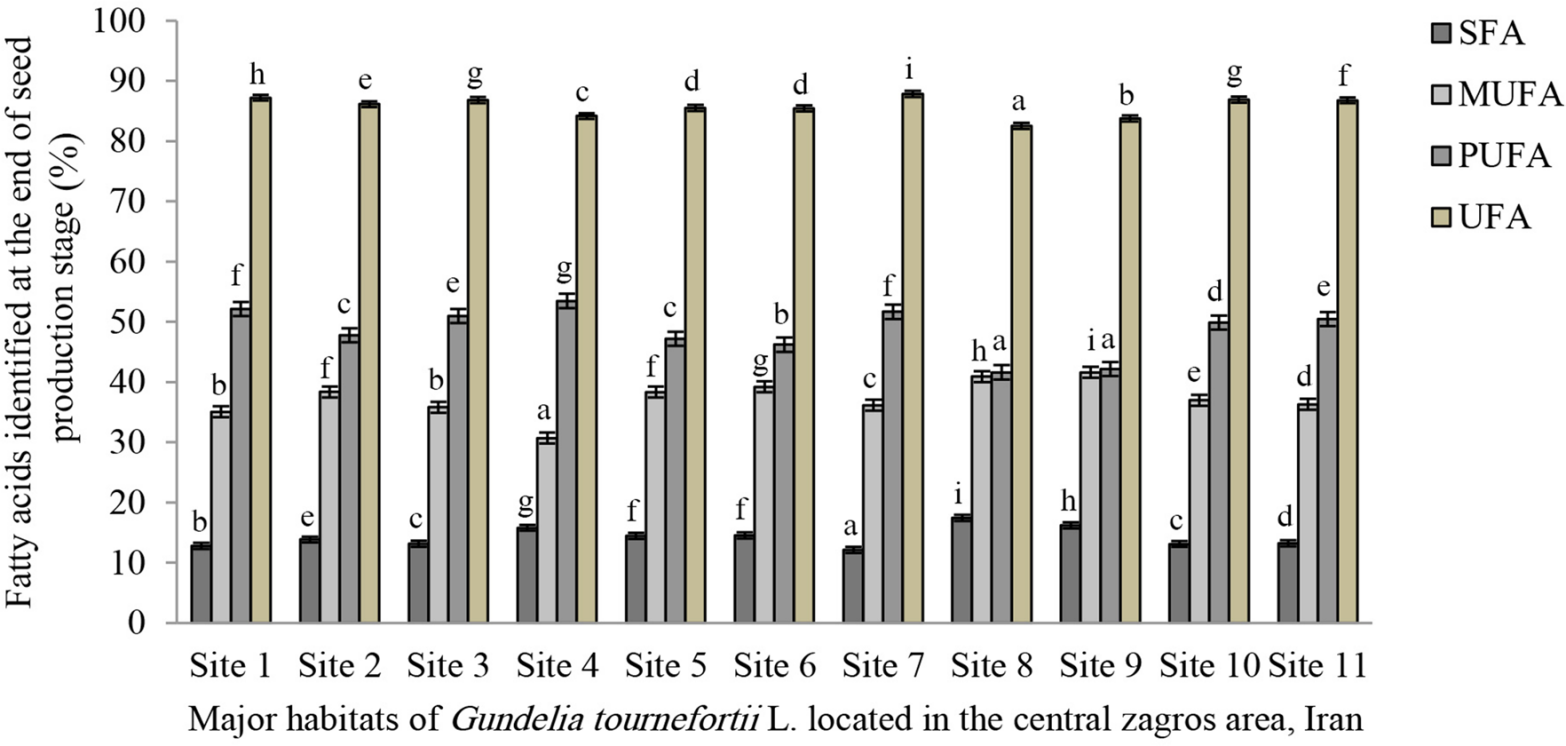
Bar chart of the quantitative amounts of SFA, MUFA, PUFA and UFA at the end of the seed production stage of *G. tournefortii* L. in the study sites.

As to the findings regarding the beginning stage, the following identified fatty acid types are tabulated in Table 4: myristic acid, palmitic acid, and stearic acid were identified as the three saturated fatty acids. The highest and the lowest volumes of myristic acid are reported in the study sites No. 2 at (0.10%) and No. 1 at (0.002%). The highest content of palmitic acid is reported in sites No. 8 at (13.50%) and No. 9 (at 12.61%). The lowest content of the mentioned compounds is recorded in site No. 7 at (9.37%). The highest volume of stearic acid is observed in sites No. 8 at (3.79%), No. 4 at (3.75%) and No. 9 at (3.61%). The lowest is reported from site No. 1 at (2.58%). The highest volumes of SFAs are reported from site No. 8 at (17.45%), site No. 9 at (16.23%) and site No. 4 at (15.80%). The lowest content of SFAs is attributed to site No. 7 at (12.15%). The highest volume of MUFAs is recorded for sites No. 9 at (41.19%), No. 8 at (40.90%) and No. 6 at (39.32%). The lowest volume is observed in site No. 4 at (30.75%). As to the PUFAs, the highest volume is attributed to linoleic acid and the lowest to linolenic acid. Sites No. 4 at (53.22%), No. 7 at (51.63%) and No. 5 at (51.23%) contain the highest volumes of linoleic acid. The lowest volume is recorded for site No. 8 at (41.33%). The highest volume of linolenic acid is obtained from sites No. 9 at (0.48%) and No. 3 at (0.39%). The lowest volume is obtained from sites No. 10 at (0.05%) and No. 11 at (0.06%). It is revealed that the highest volume of PUFAs is attributed to site No. 4 at (53.47%). The lowest volume is attributed to site No. 8 at (41.61%). The results of UFAs reveal that the highest volume of the mentioned compounds is attributed to site No. 7 at (87.83%) and the lowest amount of the mentioned compounds was reported from site No. 8 at (82.53%). The linoleic acid to linolenic acid (n-6/n-3) ratio in all sites is significantly different from each other as the highest volumes are attributed to site No. 11 at (720) and the lowest to site No. 9 at (93.51). The UFA to SFA ratio reveals that the highest volumes are attributed to site No.7 at (7.22). The lowest volume is attributed to site No. 9 at (5.15). The PUFA to SFA ratio indicates that site No. 7 at (4.25) and the lowest one is attributed to site No. 8 at (2.38). The MUFA to PUFA ratio indicates that the highest volume is of sites No. 8 and 9 both at (0.98) and the lowest is of site No. 4 at (0.57). The results of the Cox value index indicate that sites No. 4 at (5.86) and No. 7 at (5.83) have the highest volume among all sites. The lowest volume of the mentioned index is attributed to site No. 8 at (4.73). The Cox value index of the other sites fluctuates between the lowest and the highest volumes at the beginning of the seed production stage. In genera, the results obtained from Tables 3 and 4 showed that the volumes of unsaturated fatty acids of *G. tournefortii* L. seeds are much more than that its saturated fatty acids. (Figs. 1 and 2).

### Principal component analysis (PCA) and cluster analysis in the study sites

This analysis is run to assess and evaluate the volume of extract yield and the quantitative volume of the identified fatty acids and their characteristics in major habitats of *G. tournefortii* L. in both phenological stages of seed production (i.e. the beginning of the seed production and the end)^20^. Applying these techniques enables the identification of fatty acid compounds of the mentioned plant and their features in the study sites. The cluster analysis is run to assess the similarity among the study sites and their classification. These two procedures are adopted in determining the volume of extracted compounds of *G. tournefortii* L. seeds; concerning the most important environmental factors in both phenological stages. The quantitative volume of the identified fatty acids of *G. tournefortii* L. seeds and their features are classified in both phenological stages of seed production. In this study, the agglomerative hierarchical clustering process is run based on the Gower similarity index through the single linkage method introduced by 21 and 22^21,22^. The correlation matrix and its values among some important ecological variables and the volume of *G. tournefortii* L. seeds extract produced are analyzed during the two phenological stages of seed production (Supplementary Fig. 3 and Supplementary Fig. 4). The matrices and their values and features are assessed with the fatty acids identified in the study sites, too (Supplementary Fig. 5 and Supplementary Fig. 6).

PCA and cluster analysis of the extract produced volume together with major environmental factors at the beginning of seed production stage.

**Figure 3:**
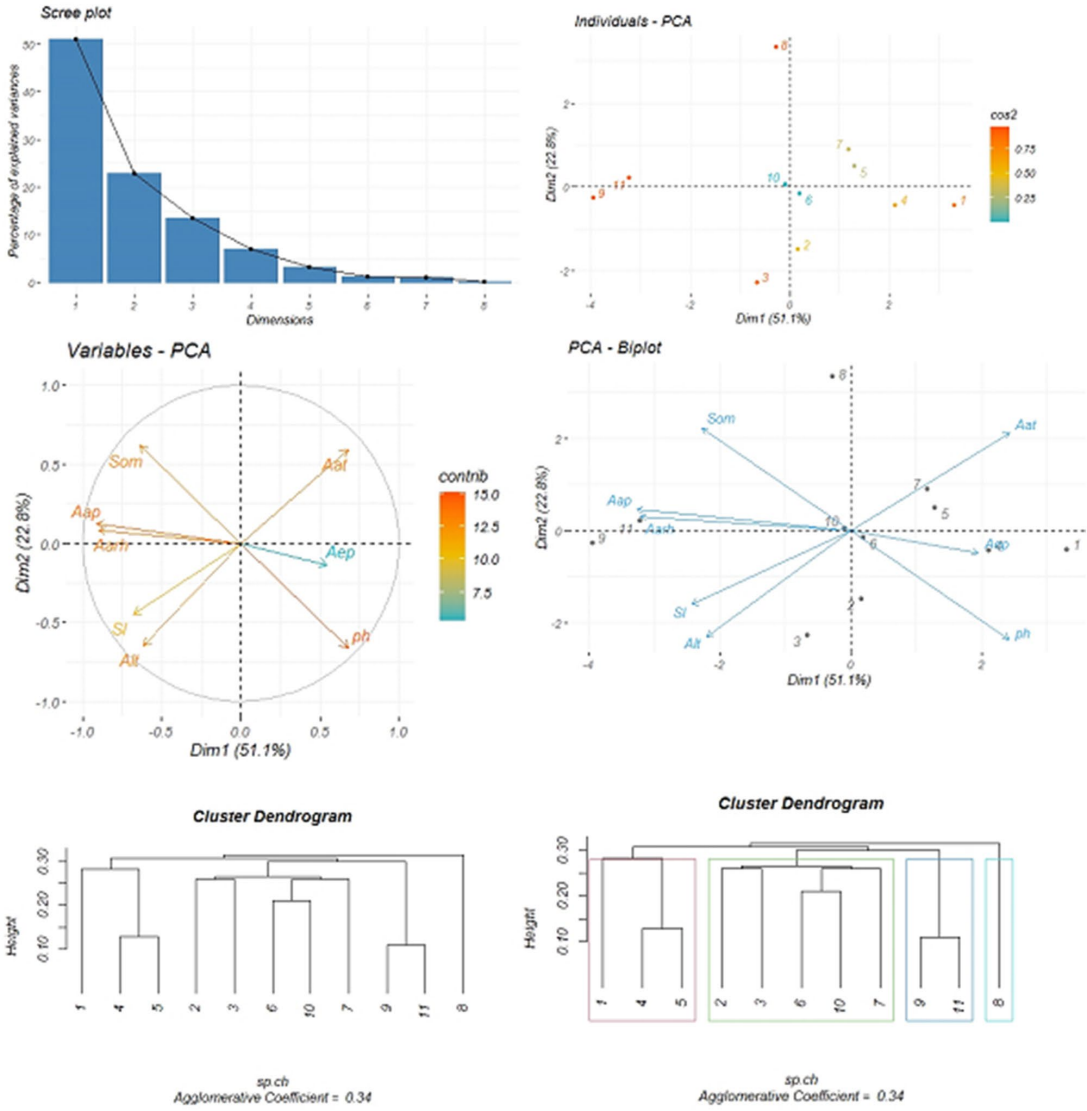
Principal component analysis (PCA) of the amount of extract produced from *G. tournefortii* L. seeds coupled with some environmental factors at the beginning of seed production stage (four graphs above) and cluster analysis based on hierarchical agglomerative clustering process using single linkage method at the beginning of seed production stage in the study sites (two below dendrograms).

At this step, the volume of extract produced by *G. tournefortii* L. seeds together with some of the major environmental ecological factors together with physiography, climate, and soil at the beginning of the seed production stage is assessed by applying the PCA method and cluster analysis. The names of some of the most important environmental factors and their calculated quantitative content on the reproductive growth stages of *G. tournefortii* L. in the study sites are tabulated in Table 9. As observed in (Fig. 3) the PC1 plotted on the horizontal axis represents the highest proportion of the variance at (51.1%), while the PC2 plotted on the vertical axis represents (22.8%). The results indicate that the volume of extract from *G. tournefortii* L. seeds is directly and positively correlated to soil pH and average annual temperature. The other environmental factors are correlated to the volume of the produced extract is correlated to the each other. The eigenvalue variance results, variance percentage, and cumulative variance percentage are tabulated in Table 5.

**Table 5:** Eigenvalue variance, variance percentage and cumulative variance percentage obtained from the amount of extract produced at the beginning of seed production stage of *G. tournefortii* L. coupled with some of the environmental factors in the study sites.

Dimension No	Eigenvalue variance	Variance (%)	Cumulative variance (%)
Dim.1	4.08	51.06	51.06
Dim.2	1.82	22.82	73.88
Dim.3	1.07	13.45	87.34
Dim.4	0.56	7.06	94.40
Dim.5	0.26	3.29	97.70
Dim.6	0.09	1.16	98.86

The results of cluster analysis indicate that the study sites are clustered in four major groups, as follows: sites No. 1, 4, and 5 constitute the first group; sites No. 2, 3, 6, 10, and 7 constitute the second group; sites No. 9 and constitute the third group. Site No. 8 constitutes the fourth group. The agglomerative coefficient is 0.34 (Fig. 3) PCA and cluster analysis of the extract produced volume together with major environmental factors at the end of seed production stage.

**Figure 4:**
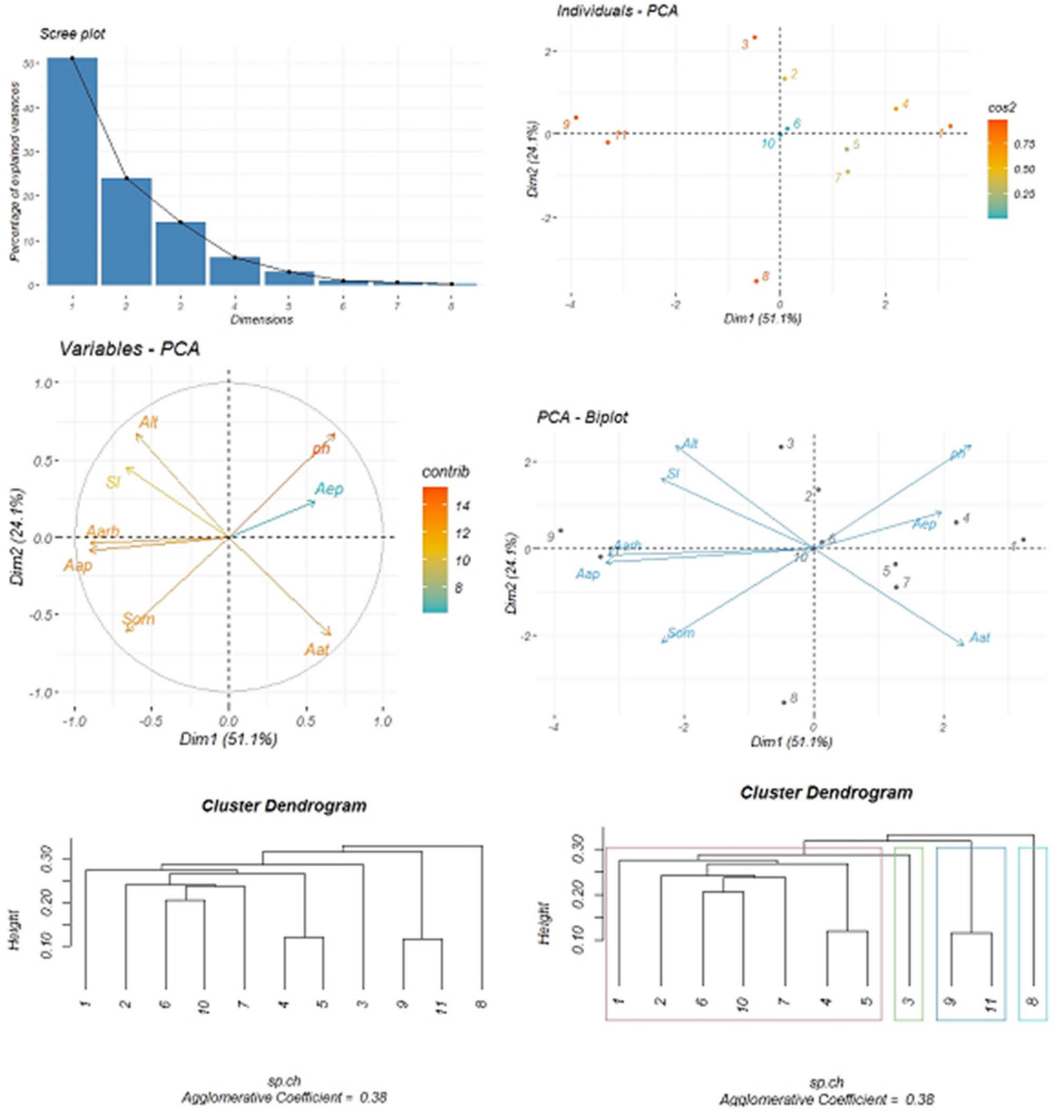
Principal component analysis (PCA) of the amount of extract produced from *G. tournefortii* L. seeds coupled with some environmental factors at the end of seed production stage (four graphs above) and cluster analysis based on hierarchical agglomerative clustering process using single linkage method at the end of seed production stage in the study sites (two below dendrograms).

The volumes of *G. tournefortii* L. seeds extract and some of the environmental factors at the end of the seed production stage are assessed by applying the PCA method and cluster analysis in this study. As observed in (Fig. 4), the PC1 plotted on the horizontal axis represents the highest proportion of the variance at (51.1%), while, the PC2 plotted on the vertical axis represents (24.1%). The results indicate a slight difference between the annual temperature and soil pH factors. A slight difference is positively and directly correlated to the yield volume subject to the geometric position of the other environmental factors regarding the two PCA dimensions. The other environmental factors are not directly correlated with the extract volume of *G. tournefortii* L. seeds, though they are correlated with each other. The results of eigenvalue variance, variance percentage, and cumulative variance percentage are shown in Table 6.

**Table 6:** Eigenvalue variance, variance percentage and cumulative variance percentage obtained from the amount of extract produced from *G. tournefortii* L. seeds at the end of the seed production stage coupled with some of the environmental factors in the study sites.

Dimension No	Eigenvalue variance	Variance (%)	Cumulative variance (%)
Dim.1	4.09	51.12	51.12
Dim.2	1.92	24.09	75.22
Dim.3	1.13	14.12	89.34
Dim.4	0.48	6.09	95.44
Dim.5	0.23	2.90	98.34
Dim.6	0.06	0.86	99.21

The results of cluster analysis indicate that the study sites are clustered in four major groups, as follows: sites No. 1, 2, 6, 10, 7, 4, and 5 constitute the first group; site No. 3 constitutes the second group; sites No. 9 and 11 constitute the third group. Site No. 8 constitutes the fourth group. The agglomerative coefficient is (0.38), (Fig. 4).

PCA and cluster analysis of the identified fatty acid compounds of *G. tournefortii* L. seeds and their features at the beginning of the seed production stage.

The identified fatty acids compounds of *G. tournefortii* L. seed and their relevant features at the beginning of seed production are assessed by applying the PCA method and cluster analysis. The PC1 plotted on the horizontal axis represents the highest proportion of the variance at (53%), while the PC2 plotted on the vertical axis is at (41.9%) of the total variation. The six identified fatty acid compounds, are correlated and displayed in two PCA dimensions. Myristic acid is positively correlated with palmitic and linolenic fatty acids, with a negative correlation with oleic and stearic fatty acids. There exists no significant correlation between myristic acid and linoleic acid. Palmitic acid is positively correlated with linolenic acid; while there exists no strong correlation between palmitic acid and the other three fatty acids. A significant and strong correlation is observed between stearic acid and oleic acid, however, a moderate correlation is observed between stearic, linoleic, and linolenic acids. A relatively strong correlation is observed between oleic and linolenic fatty acids, however, as mentioned above, the identified fatty acids, as one or in combination, have different degrees of correlation. The features of the identified fatty acids according to PCA reveal that the lowest contribution of the studied variables is attributed to the linoleic acid to linolenic acid ratio. The other variables indicate a higher contribution in the PCA dimensions. Therefore, the highest correlation among saturated fatty acids is attributed to palmitic acid. The highest correlation among the MUFAs is allocated to oleic acid. The highest correlation among PUFAs is attributed the linoleic acid. The correlation between SFAs and UFAs is observed in their negative and inverse sense. The UFAs to SFAs ratio indicates a negative and inverse correlation with the SFAs, while the same is a positive and direct correlation in the UFAs. This ratio is negatively and inversely correlated with palmitic acid. PUFAs to SFAs ratio indicate that there exists a negative and direct correlation with SFAs. On the contrary, the UFAs to SFAs ratio are positively and directly correlated. The result of the MUFAs to PUFAs ratio indicates a positive and direct correlation with the amount of MUFAs and a negative and inverse correlation with the PUFAs volume. Moreover, a positive and direct correlation is observed between this ratio and the fatty acid compounds the oleic and stearic fatty acids. The eigenvalue variance, variance percentage, and cumulative variance percentage are shown in Table 7.

**Table 7:** Eigenvalue variance, variance percentage and cumulative variance percentage obtained from the identified fatty acids compounds of *G. tournefortii* L. seeds and its features at the beginning of the seed production stage in the study sites.

Dimension No	Eigenvalue variance	Variance (%)	Cumulative variance (%)
Dim.1	7.41	52.97	52.97
Dim.2	5.86	41.91	94.89
Dim.3	0.57	4.13	99.02
Dim.4	0.07	0.56	99.58
Dim.5	0.03	0.21	99.80
Dim.6	0.01	0.11	99.92

**Figure 5:**
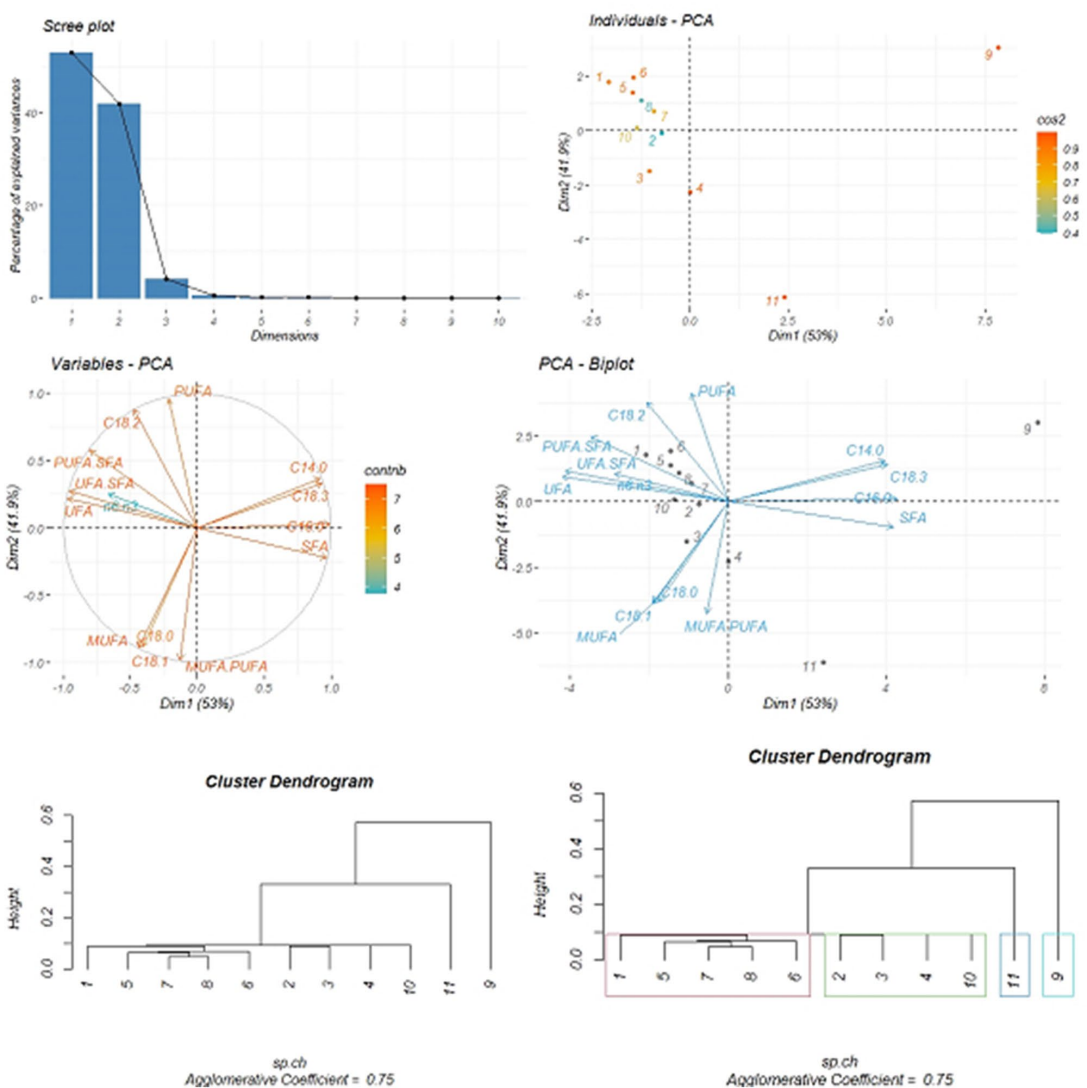
Principal component analysis (PCA) of the fatty acid profiles of *G. tournefortii* L. seeds and their features at the beginning of seed production stage (four graphs above) and cluster analysis based on hierarchical agglomerative clustering process using single linkage method at the beginning of seed production stage in the study sites (two below dendrograms).

The results of cluster analysis indicate that the study sites are clustered in four major groups, as follows: sites No. 1, 5, 7, 8, and 6 constitute the first group; sites No. 2, 3, 4, and 10 constitute the second group. Site No. 11 constitutes the third group. Site No. 9 constitutes the fourth group. The agglomerative coefficient is 0.75 (Fig. 5).

PCA and cluster analysis of the fatty acid compounds of *G. tournefortii* L. seeds and their features at the end of seed production stage.

The fatty acid compounds of *G. tournefortii* L. seeds and their relevant features at the end of the seed production stage are analyzed through the PCA method and cluster analysis. The PC1 plotted on the horizontal axis represents the highest proportion of the variance at (67%), while PC2 plotted on the vertical axis is (18.3%) of the total variation. The correlation between fatty acid compounds is assessed followed by determining the correlation among their related properties with each other and with fatty acid compounds the following results: The myristic acid is negatively and moderately correlated with stearic acid; the correlation between palmitic acid and the other three stearic, linoleic and linolenic fatty acid compounds, are reported positive and very strong, negative and strong and positive and moderate, respectively. In this process, a significant and strong correlation is observed between stearic and palmitic acids. Stearic acid is negatively and moderately correlated with linoleic acid. Oleic acid is negatively and inversely correlated with linoleic acid. There exists a positive and direct correlation between linoleic acid, palmitic, and linolenic fatty acids, while linoleic acid is negatively correlated with oleic acid and stearic acids. The linoleic acid is negatively correlated with palmitic acid, with a negative correlation with oleic acid and a moderate correlation with linolenic acid. Linolenic acid is positively and moderately correlated with palmitic acid and negatively and inversely correlated with linoleic acid. The myristic and linolenic compounds fatty acids have a lower contribution in PCA dimensions compared to other compounds. The results obtained from the features of the identified fatty acids by applying the PCA method are: the lowest contribution of the features belongs to the linoleic acid to the linolenic acid ratio in the PCA dimensions and the highest correlation among SFAs is attributed to palmitic and stearic acids. The highest correlation among the MUFAs monounsaturated fatty acids is attributed to oleic acid. The highest correlation among PUFAs is attributed to linoleic acid. The correlation between SFAs and UFAs is negative and inverse. In this context, the palmitic and stearic fatty acids are negatively and inversely correlated with the ratio UFAs to SFAs ratio. There exists a negative and inverse relation between SFAs and UFAs ratio. This ratio is negatively and inversely correlated with SFAs and it is positively and inversely correlated with the two other features of the same fatty acids. This ratio is negatively and inversely correlated with the palmitic and stearic fatty acids. This ratio is positively and directly correlated with linoleic acid. The MUFAs to PUFAs ratio indicates a positive and direct correlation with MUFAs volume and is negatively and inversely correlated with the PUFAs. This ratio is positively and directly correlated with oleic acid and negatively and inversely correlated with linoleic acid. The eigenvalue variance, percentage variance percentage, and cumulative variance percentage are tabulated in Table 8.

**Table 8:** Eigenvalue variance, variance percentage and cumulative variance percentage obtained from the identified fatty acids compounds of *G. tournefortii* L. seeds and their features at the end of seed production stage in the study sites.

Dimension No	Eigenvalue variance	Variance (%)	Cumulative variance (%)
Dim.1	9.38	67.04	67.04
Dim.2	2.56	18.30	85.34
Dim.3	1.42	10.18	95.53
Dim.4	0.49	3.56	99.09
Dim.5	0.09	0.64	99.74
Dim.6	0.01	0.14	99.88

**Figure 6:**
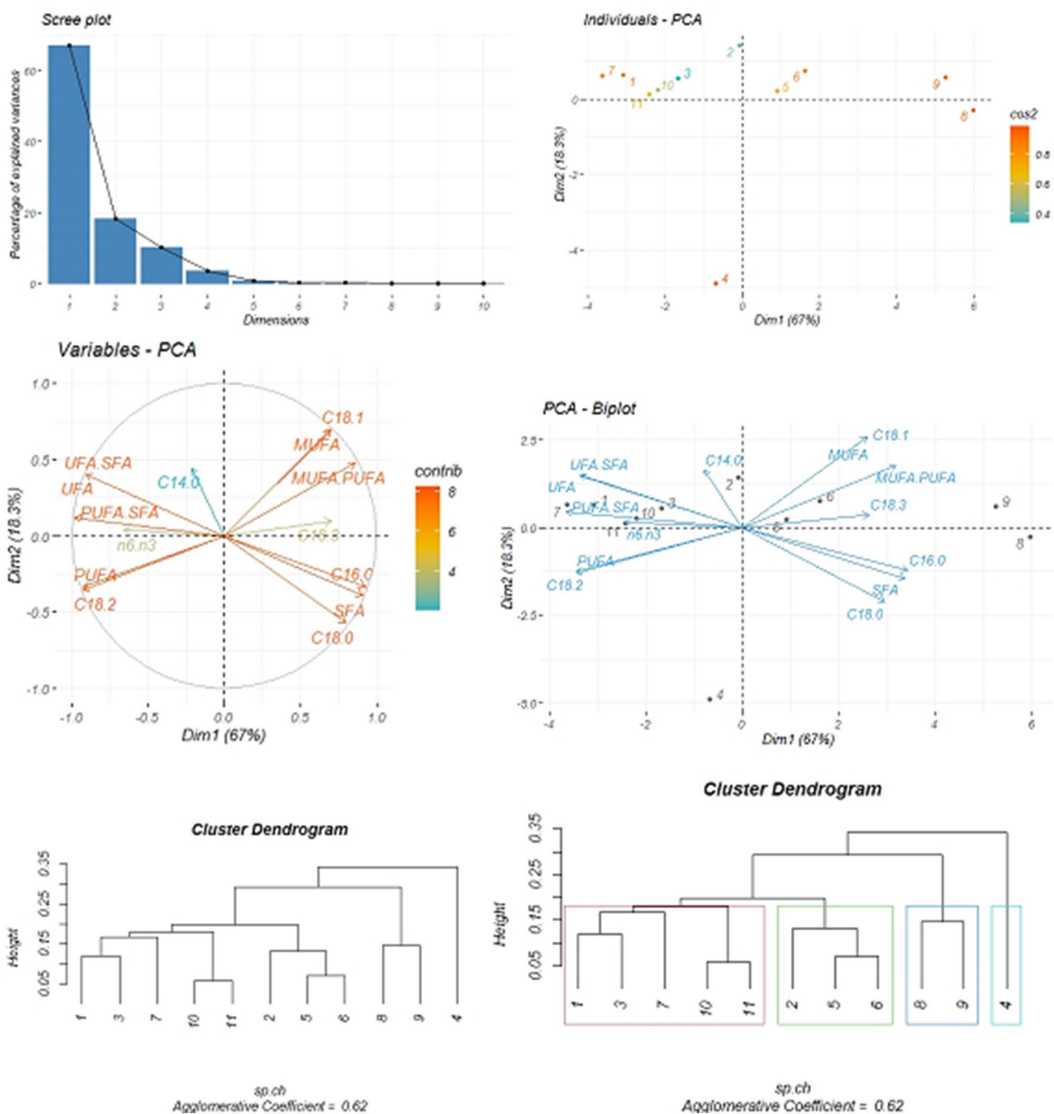
Principal component analysis (PCA) of the fatty acid profiles of *G. tournefortii* L. seeds and their features at the end of seed production stage (four graphs above) and cluster analysis based on hierarchical agglomerative clustering process using single linkage method at the end of seed production stage in the study sites (two below dendrograms).

The results of cluster analysis indicate that the study sites are clustered in four major groups, as follows: sites No. 1, 3, 7, 10, and 11 constitute the first group; sites No. 2, 5, and 6 constitute the second group; sites No. 8 and 9 constitute the third group. Site No. 4 constitutes the fourth group. The agglomerative coefficient is 0.62 (Fig. 6).

## Discussion

Plant seed, grown in the wilderness is an important source of oil for nutritional, medicinal, and industrial use in natural areas. As different oil sources have different compositions, seeking to introduce new oil sources for nutrition to generate energy and assure health is necessary and inevitable. All plant organs of *G. tournefortii* L. (roots, stems, leaves, flowers, and seeds) are consumed^23^. The genera of *Gundelia* L. originate in the Middle East and Mediterranean regions, among which *G. tournefortii* L. is well known and specified as a valuable food source^24^. The constituent elements of this plant are applied for the treatment of different diseases like diarrhea and bronchitis, skin diseases, pain, diarrhea, respiratory diseases, digestive disorders, laxatives, sedatives, stroke, gastric ailments, hypoglycemic, vitiligo, high blood pressure and cancer^25–33^.

The seed oil extract of *G. tournefortii* L. is reported at the end of the seed production stage in all study sites. Site No. 6 is an exception as its yield extract at the beginning of the seed production stage is higher than that of the stage. The fatty acid profiles of *G. tournefortii* L. are not altered qualitatively at both phenological stages of seed production, while different volumes are recorded quantitatively in the study sites. In both phenological stages of seed production, six fatty acids compounds are identified each with a different range of volume in all study sites. Nine specific and identical features are considered and assessed for both phenological stages of seed production. Among these features, the highest volume of the reported variations at both phenological stages of seed production belongs to the linoleic acid to linolenic acid ratio, while the lowest volume is attributed to the Cox value index among the study sites. In general, the unsaturated fatty acids volume is recorded higher than that of the SFAs at both phenological stages of seed production. Moreover, the volume of SFAs at the end of the seed production stage is higher than that at the beginning stage. The volume of PUFAs is higher than that MUFAs in both seed production stages. The daily consumption of n-3 PUFAs in the diet is important as they have many beneficial effects on the physiological functions of the human body, like blood pressure, heart rate, triglycerides, inflammation, endothelial function, and cardiac diastolic^34^. In general, oily fish types are like tuna, salmon, mackerel, herring, and sardines most important sources of n-3 PUFAs^35^. Likewise, there exists a substantial volume of Linoleic acid (LA, n-6 PUFA) in many vegetable oil types like sunflower, soybean, corn, and grape seeds^35^. Linoleic acid is also found in some products processed from these oil types, like margarine^35^. Considerable, volumes of alpha-Linolenic acid (ALA, n-3 PUFA) are found in many plant sources. Some well-known and common plant oil sources include soybean and rapeseed, vegetable, some nuts, and above all, linseeds and linseed oil types^35^. The World Health Organization has focused on the LA to ALA in diet ratio^36^; Consequently, the minimum intake level for EFA should be (2.5%) LA and (0.5%) ALA to prevent deficiency symptoms and provide the necessary energy for adults^37^.

There exists only one article in Farsi, where some ecological factors affecting the vegetative growth stage of *G. tournefortii* L., and the fatty acid compounds extracted are assessed^39^. Matthaus and Ozcan (2011) there exist were seven fatty acid compounds in the extracted oil from *G. tournefortii* L. where linoleic and oleic fatty acids yield (57.8%) and (28.5%) as potential nutrient sources, respectively ^33^. The findings of this study correspond with that of 33. Abdul et al. (2012) run a study on the fatty acids content in the *Gundelia* L. oil where eight fatty acids in *G. tournefortii* L. seed with high oleic acid and linoleic acid content at (40.13%) and (20.33%), respectively^40^. Because oleic acid is identified as the first compound in their study, it does not correspond with the results of this study. Different quantitative volumes are reported on other identified compounds in the study, which do not correspond with this study. Khanzadeh et al. (2014) run a study on the physiochemical properties of *G. tournefortii* L. seed oil and identified 11 fatty acids. Three of these fatty acid types are linoleic acid, oleic acid, and palmitic acid with volumes of (54.59%), (29.59%) and (9.88%) predominant compounds, respectively^41^. The compounds identified in their study are consistent with the ones at different volumes. Zarei et al. (2013) assessed some of the ecological features and seed content of *G. tournefortii* L., where the *G. tournefortii* L. had an appropriate growth potential condition with some of the ecological factors like average annual rainfall (241.8 mm), average annual temperature (18 °C), soil pH (8.18) and soil EC (1.3 ds/m) in the mentioned area. They confirmed that *G. tournefortii* L. seeds contain 10 fatty acids. Among these 10, three compounds of linoleic acid (45.46%), oleic acid (38.5%), and palmitic acid (10.42%) are outstanding^39^. Their results in both ecological characteristics and fatty acids sections are consistent with the results of this study. Al-Saadi et al. (2017) assessed the variation in fatty acid methyl ester contents and composition and found three fatty acid compounds in *G. tournefortii* L. oil seeds, where the highest volumes are recorded as linoleic acid (43.98%), oleic acid (28.29%) and palmitic acid (13.42%), respectively^42^. Their obtained results correspond with the results of this study.

From a general overview, the cluster analysis is run on quantitative plant ecology and in a wide range of other scientific fields. This analysis is run to find pattern and order in a data set where a series of groups is found with the volume of variance within groups being at its minimum and between groups at its maximum^43^. The results of cluster analysis in different sections of results revealed that the study sites based on the existence of similarities among them can be classified into different clusters and separated from each other.

As to the isolation and identification of secondary metabolites of medicinal plants, due to the presence of beneficial bioactive compounds controlled by genetic processes and influenced by environmental factors, this issue has always been and is a concern by the involved researchers. It is suggested that the role of physical properties and morphological features of *G. tournefortii* L. seeds, together with relevant supplementary studies on its genetic diversity, be assessed combined. Moreover, considering the two agronomic factors of *G. tournefortii* L. namely breed improvement and crop improvement at the farmland scale, together with assessing the ecological features of its wild cultivars will yield more realistic results.

## Methods

### Reagents (solvent and chemicals)

The fatty acids reference samples are the myristic acid (C14:0; tetradecanoic acid), palmitic acid (C16:0; hexadecanoic acid), stearic acid (C18:0; octadecanoic acid), oleic acid (C18:1; 9-octadecenoic acid), linoleic acid (C18:2; 9,12–octadecadienoic acid) and linolenic acid (C18:3; 9,12,15–octadecatrienoic acid), purchased from Sigma-Aldrich (St. Louis MO). Petroleum ether (40–60C) was purchased from Merck chemical company, Germany (purity > 98%) for seed oil extraction. Natrium methylate (CH3ONa) is purchased from Merck Company (Schuchardt Germany). Methanol extra pure (CH_3_OH, purity ≥ 99.9%) and *n*-Hexane (C6H14) analytical grade is purchased from Merck chemical company (Darmstadt Germany). Sodium sulfate (Na_2_So_4_) is purchased from Aldrich (Munich Germany). Silicon grease (Loxeal Cesano M. Italy) is purchased for the experiment.

### Studied sites and some of its ecological features

**Figure 7:**
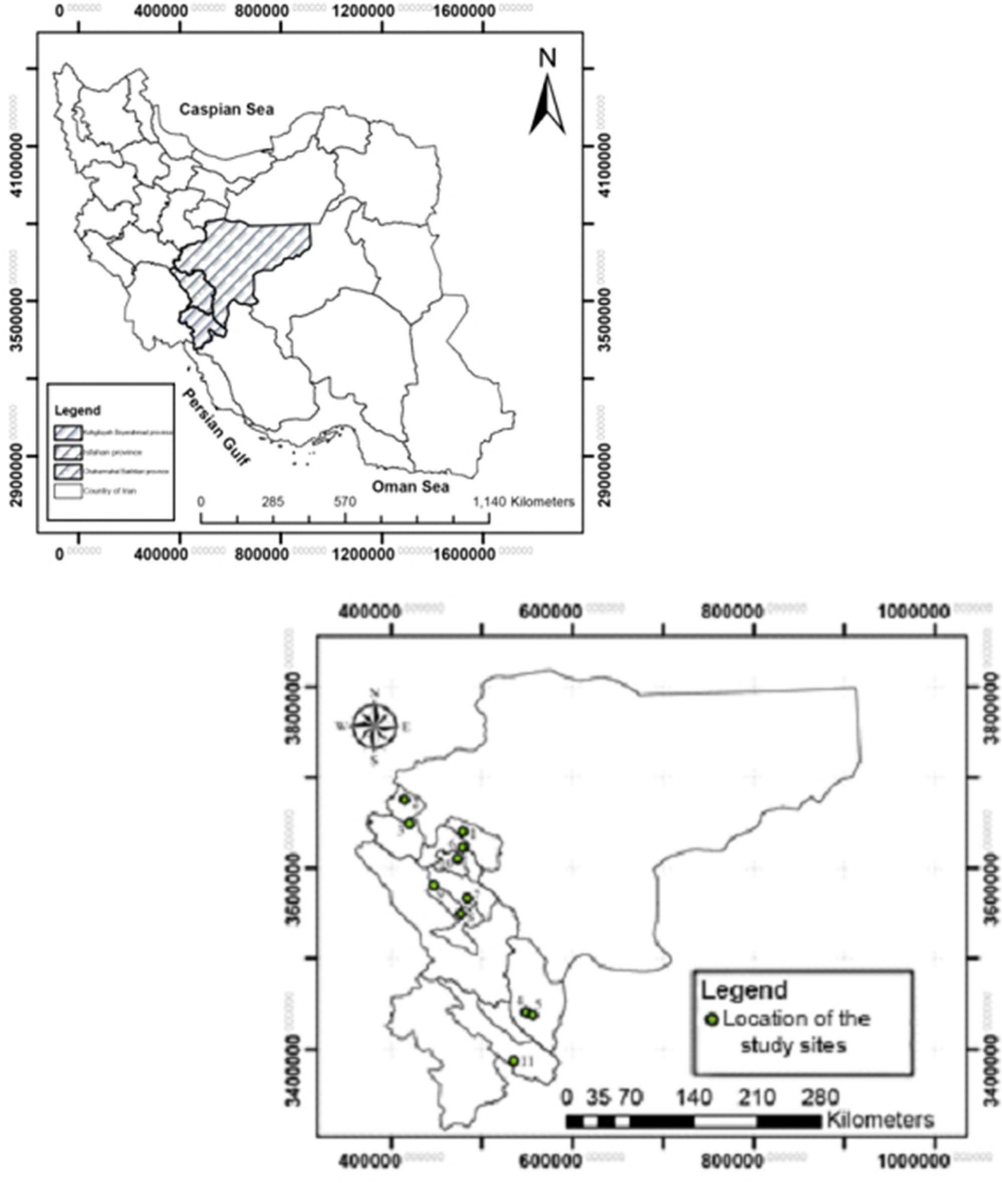
Geographical location of study areas in the Central Zagros region of Iran (points marked on the map indicate the study sites).

The Central Zagros region of Iran covers about three million and one hundred thousand hectares, considered a significant research and economic pole in terms of cultivation, production, and medicinal plants processing. The existence of the factors like rich biodiversity, specific climatic conditions, diverse mountainous areas, many watersheds and rivers, and fields covered by forests and rangelands are the prominent features of this region. In general, 11 major habitats of the *G. tournefortii* L. plant are selected as different studied sites (Fig. 7). Some of the influential ecological features on the reproductive growth of the mentioned plant are assessed and determined. (Table 9).

**Table 9:** Some of the most important ecological features in the study sites.

Samples	Geographical coordinates		Major ecological features								Climate (Demartonne method)
	Longitude	Latitude	Altitude (m)	Slope (%)	AAP* (mm)	AAT* (°C)	AARH* (%)	Som* (%)	pH	Ap* (mg/kg)	
Site 1	50° 47′ 29.3″ E	33° 54′ 35.7″ N	2215	8.9	250.4	14.3	27.4	0.31	7.81	574.54	Semi-arid
Site 2	50° 05′ 47.7″ E	33° 12′ 58.9″ N	2259	35.92	378.9	10.6	31.5	0.63	7.78	372.24	Semi-arid
Site 3	50° 13′ 34.5″ E	32° 57′ 20.7″ N	2456	29.75	524.8	9.5	35.2	0.41	7.74	790.58	Sub-humid
Site 4	51° 32′ 11.2″ E	31° 08′ 40.3″ N	2085	10.13	393.3	11.6	32.1	0.33	7.68	763.08	Semi-arid
Site 5	51° 35′ 42.5″ E	31° 05′ 20.3″ N	2113	9.4	398.9	11.3	32.7	0.87	7.63	831.82	Semi-arid
Site 6	50° 46′ 45.3″ E	32° 44′ 10.2″ N	2377	30.19	309.3	11.8	29.6	0.92	7.52	167.43	Semi-arid
Site 7	50° 50′ 23.6″ E	32° 12′ 41.3″ N	1958	28.4	354.9	13.5	30.08	0.64	7.59	321.40	Semi-arid
Site 8	50° 45′ 46.3″ E	32° 02′ 44.4″ N	1924	9.7	562.4	13.8	35.5	1.1	7.35	553.44	Mediterranean
Site 9	50° 26′ 41.6″ E	32° 21′ 31.2″ N	2480	39.8	789.9	9.8	41.9	0.95	7.41	288.23	Very humid a
Site 10	50° 43′ 14.1″ E	32° 37′ 29.7″ N	2342	19.4	414.2	10.8	30.5	0.81	7.47	492.66	Semi-arid
Site 11	51° 22′ 26.6″ E	30° 36′ 57.9″ N	2463	28.67	671.4	9.9	40.7	0.99	7.38	323.68	Sub-humid

*AAP* average annual precipitation, *AAT* average annual temperature, *AARH* average annual relative humidity, *Som* soil organic matter, *AP* Absorbable potassium.

### Plant material

*G. tournefortii* L. is a perennial spiny native plant grown extensively in the central Zagros region of Iran between March and April. *G. tournefortii* L. has large and vertical roots and semi-grass and branched stems, splitting into a flower. Its leaves embrace and surround the stem, without petioles, ending with deep cuts and jagged edges. The plant seeds are light and elongated with hairy umbrellas, with a very high ability in viability^14^. In general, the rangeland ecosystems are parts of watersheds managed by the Ministry of Agricultural Jahad of Iran. To run this study, the necessary coordination is made with the authorities to collect the mentioned plant, subject to permission from the Natural Resources and Watershed Management Organization of Iran a subsidiary of the Ministry of Agricultural Jahad of Iran through letter Number 121/99/6778 dated May 31, 2020. The taxonomic identity of the mentioned plant is confirmed by comparing the collected voucher specimen with that of the known identity available in the herbarium of the Department of Natural Resources, Isfahan University of Technology, Iran. The collected specimens of the *G. tournefortii* L. plant are matched with their Voucher specimens number HIUT6171 in the herbarium of the Department of Natural resources by Mrs. Mahnaz Bayat, the official herbarium botanist expert of the Department of Natural Resources at the Isfahan University of Technology, Iran (her Email address is m.bayat@of.iut.ac.ir).

### Sample preparation

**Figure 8:**
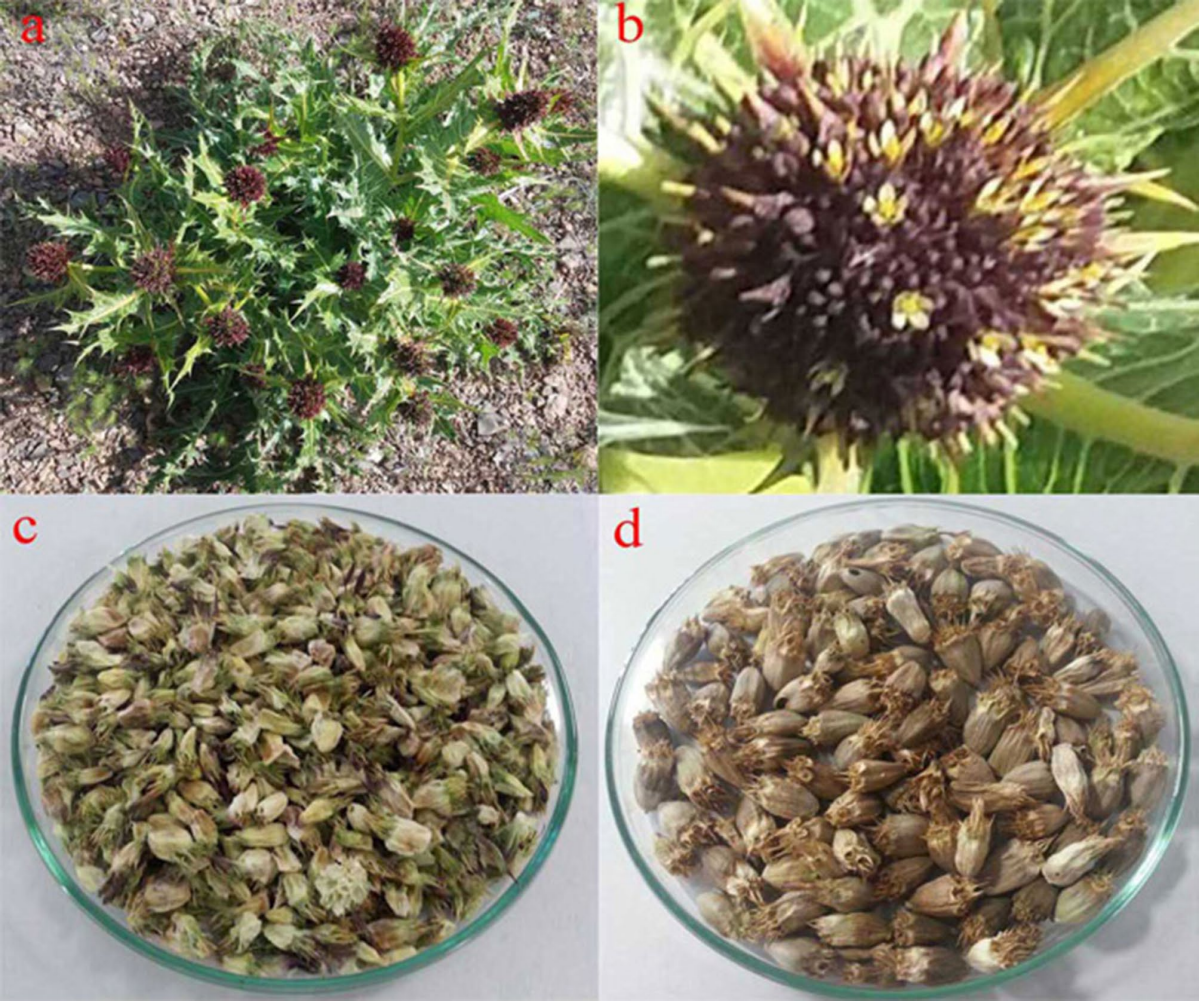
Part (**a**): *Gundelia tournefortii* L.; Part (**b**): The capitol inflorescence of *G. tournefortii* L.; Part (**c**): *G. tournefortii* L. seeds samples the beginning of seed production stage; Part (**d**): *G. tournefortii* L. seeds samples at the end of seed production stage.

First, the phenological study of the reproductive stage of *G. tournefortii* L. seeds is assessed and analyzed during the two different periods, the beginning of seed production and the end of seed production (Fig. 8). The sampling process is run based on a completely randomized design by applying the transect quadrat method in all study sites at the beginning and end of seed production stages (Table 10). Next, the sample size is determined in each study site according to 15^15^, where, the flower buds of *G. tournefortii* L. are clipped in sampling units and placed in specific sampling bags and then, the samples are transferred to the botanical laboratory of the Department of Natural Resources at Isfahan University of Technology, Iran. The collected seeds are dried in a standard situation without light, infection, and humidity within 21 days and the incomplete and immature samples are separated initially. The dried seeds are milled into smaller pieces through an electric mill (Model PX-MFC90D). The samples are separated and packed from two phenological stages of seed production.

**Table 10:** General specifications of vegetation sampling in the major habitats of *G. tournefortii* L. in the central Zagros region, Iran.

Site No	Sample size (number of quadrat)	Number of line transects	Transect distances (m)	Quadrat distances on transect (m)	Transect length (m)
Site 1	90	3	50	10	300
Site 2	135	3	50	10	450
Site 3	120	3	50	10	400
Site 4	90	3	50	10	300
Site 5	90	3	50	10	300
Site 6	120	3	50	10	400
Site 7	120	3	50	10	400
Site 8	90	3	50	10	300
Site 9	135	3	50	10	450
Site 10	90	3	50	10	300
Site 11	120	3	50	10	400

### Extraction of the *G. tournefortii* L. seeds oil

100 g of the milled samples of *G. tournefortii* L. seeds are consumed for seed oil extraction. Petroleum ether (40–60 °C) solvent is consumed for the seed oil extraction through the Soxhlet apparatus for 5 hours^16^. After the oil and solvent mixture is filtered through Whatman No. 1 filter paper. After that, the solvent is removed by a rotary vacuum evaporator (Model IKA HB 10) and the yield oil is kept in the refrigerator at 4 °C for further examination. This experiment is run separately for each phonological stage.

### Fatty acid compounds and chromatographic conditions

To determine fatty acids profiles of *G. tournefortii* L. seed oil, first, the samples are initially methylated according to the AOAC method^17^, Next, the methylated samples (1μL) are injected into the gas chromatograph (BEIFEN 3420A) equipped with Flame Ionization Detector (FID), and then, the fatty acid methyl esters of each sample are separated through HP-88 fused silica WCOT (100 m × 0.25 mm × 0.20 μm). Nitrogen is consumed as a carrier gas with a 0.5 ml/min flow rate. The temperature program of this column is adjusted as: first, the column is kept at 175 °C for one min, and next the temperature is increased to 240 °C for 2.5 min. The total time recorded is 29 min. The injection temperature is 250 °C with a 1:30 split ratio.

### Statistical analyses

The analysis is run performed for all major habitats, including the quantitative and qualitative volume yield extract and fatty acid profiles for each sample during the two phenological stages. In this context, the results are reported as Mean ± SD with replicate analysis (n = 3) by SPSS statistical software version 21. The R statistical software version 4.0.4. is applied to run the PCA and cluster analysis. All the ("Reshape2"), ("ade4"), ("ggplot2"), ("factoextra"), ("lattice"), ("permute"), ("vegan"), ("cluster") and ("tidyverse") packages applied in R Studio software are named (programming language for calculations and visual images obtained through computer processing).

## Supplementary Information

Supplementary Information.

## Data Availability

Due to privacy and ethical concerns, the data and material of the current study are available from the corresponding author on reasonable request.

## Code availability

The datasets generated during and analyzed during the current study are available from the corresponding author on reasonable request.
