# Supplementary material for: Spatio-temporal variations of extract produced and fatty acid compounds identified of Gundelia tournefortii L. seeds in central Zagros, Iran
Source: Sci Rep. 2023 May 11;13:7665. doi: 10.1038/s41598-023-34538-5 (PMC10175287; doi:10.1038/s41598-023-34538-5)
Supplement: Supplementary file 1 — Supplementary Information. [file 41598_2023_34538_MOESM1_ESM.docx]

**Title**:

**Spatio-temporal variations of extract produced and fatty acid compounds identified of *Gundelia tournefortii* L. seeds in central Zagros, Iran**

**Author names and affiliations:**

**H.R. Karimzadeh^1, *^, H.R. Farhang^1^, M. Rahimmalek^2^, M. Tarkesh Esfahani^1^**

^1^Department of Natural Resources, Isfahan University of Technology, 84156–83111 Isfahan, Iran

*** Corresponding author.**

E–mail address: [karimzadeh@iut.ac.ir](mailto:karimzadeh@iut.ac.ir); Tel.: +98 313 3913558; Fax.: +98 313 3912840

**Present/permanent address:**

***Corresponding author address.** Department of Natural Resources, Isfahan University of Technology, 84156–83111 Isfahan, Iran

^2^Department of Horticulture, College of Agriculture, Isfahan University of Technology,
84156–83111 Isfahan, Iran

**
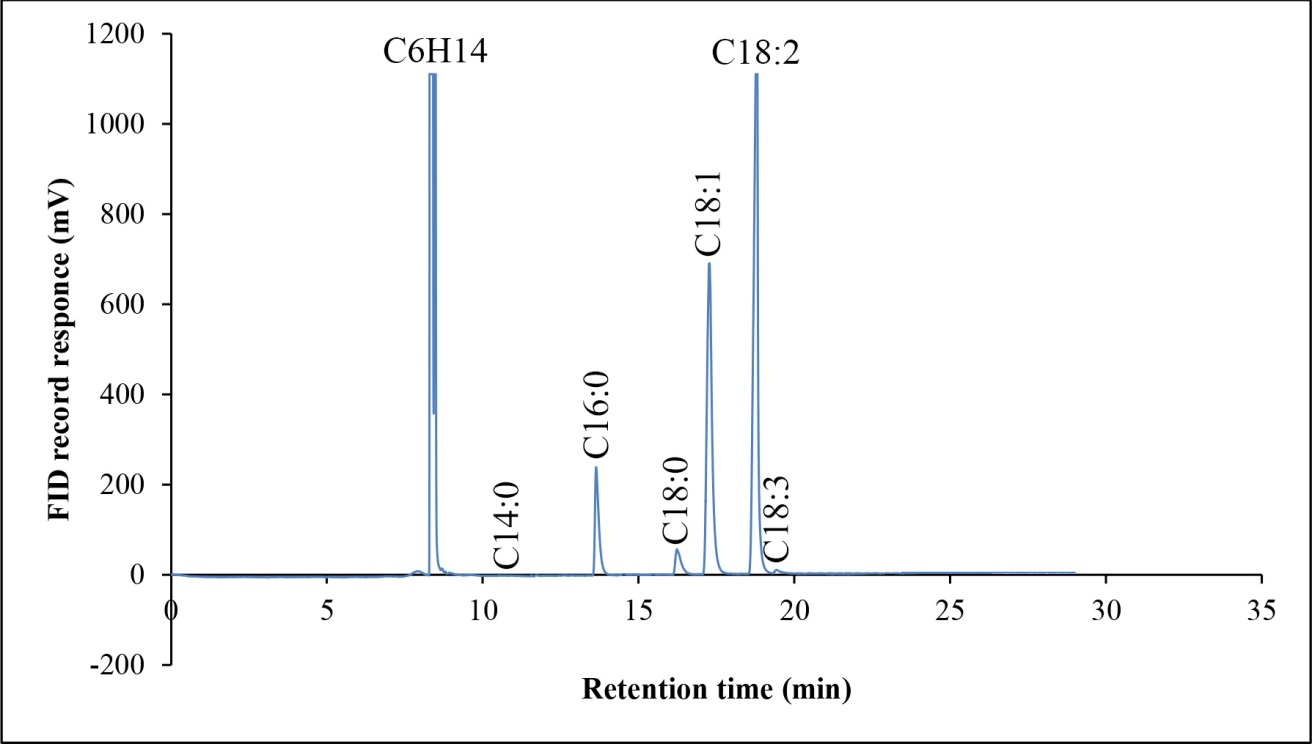
**

**Fig. S1.** GC-FID chromatogram of the identified fatty acids of *G. tournefortii* L. seeds at the beginning of seed production stage.

**
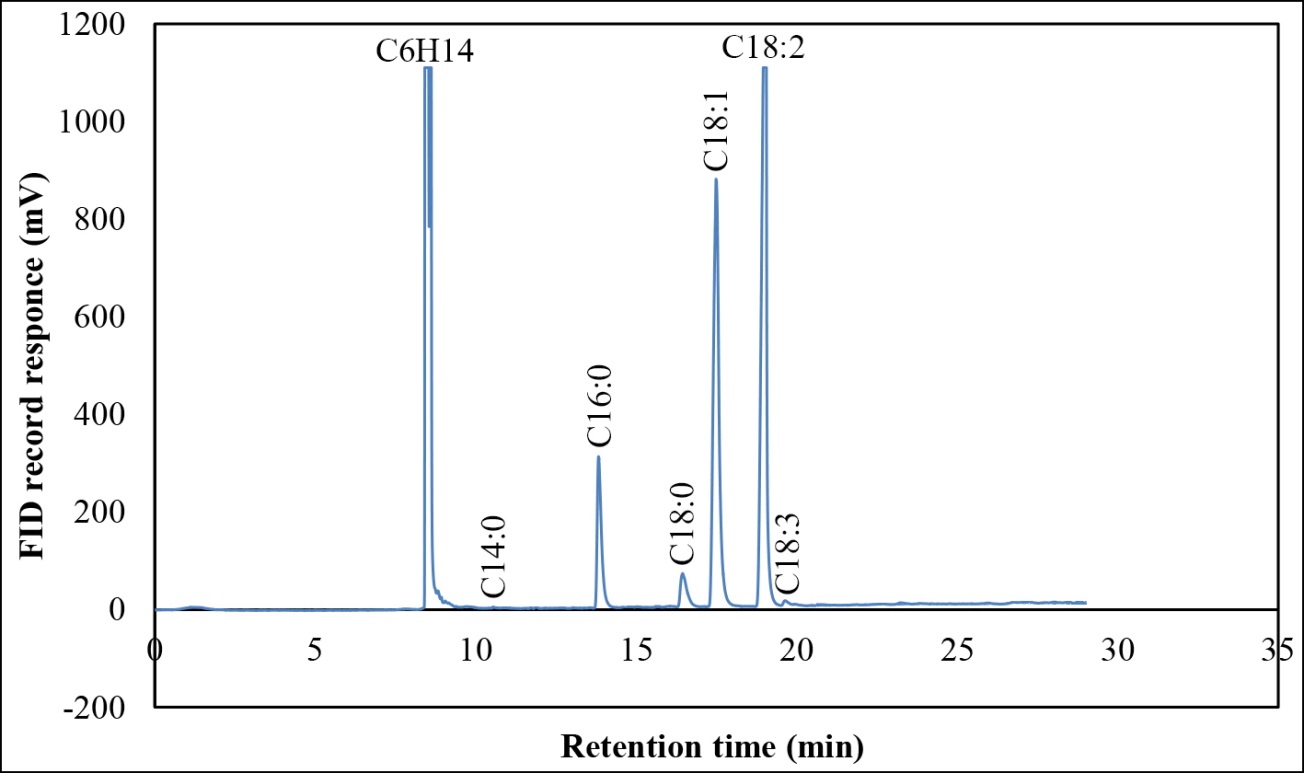
**

**Fig. S2.** GC-FID chromatogram of the identified fatty acids of *G. tournefortii* L. seeds at the end of seed production stage.


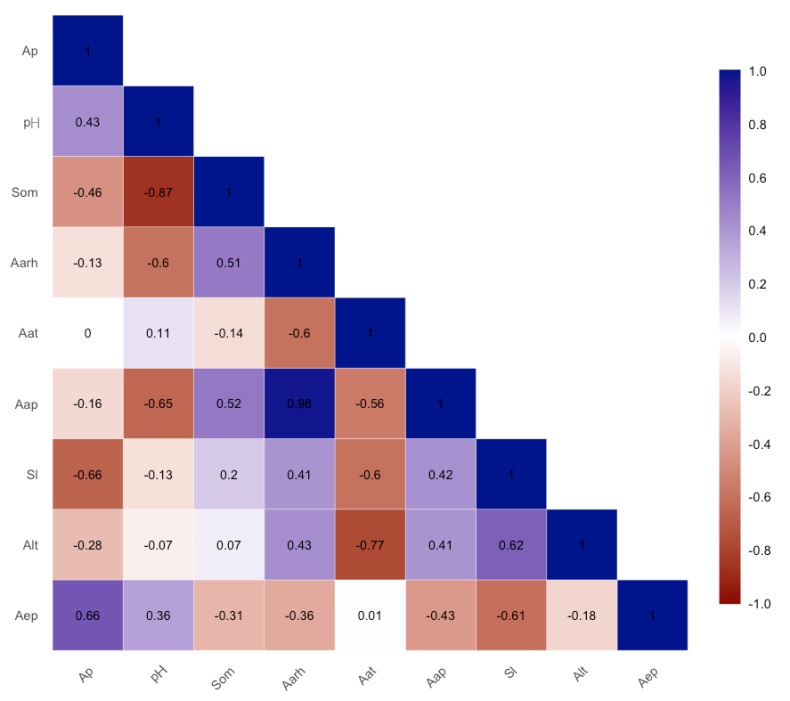


**Fig. S3.** Pearson correlation analysis of the amount of extract produced from *G. tournefortii* L. seeds (Aep) and eight major environmental factors at the beginning of seed production stage. (The names of some of the most important environmental factors and their calculated quantitative content on the reproductive growth stage of *G. tournefortii* L. in the study sites has already reported in Table 9 of main file of the manuscript).


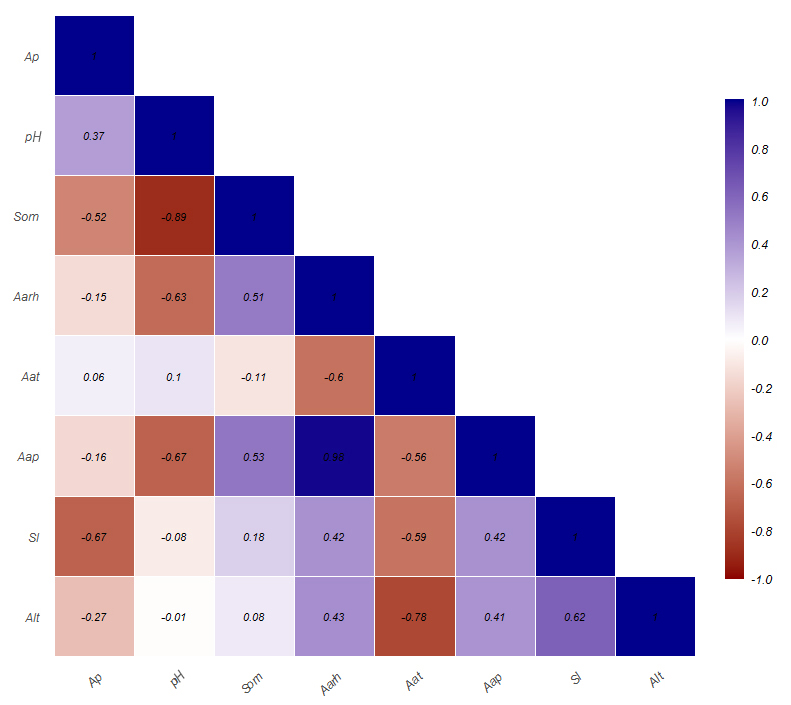


**Fig. S4.** Pearson correlation analysis of the amount of extract produced from *G. tournefortii* L. seeds (Aep) and eight major environmental factors at the end of seed production stage.


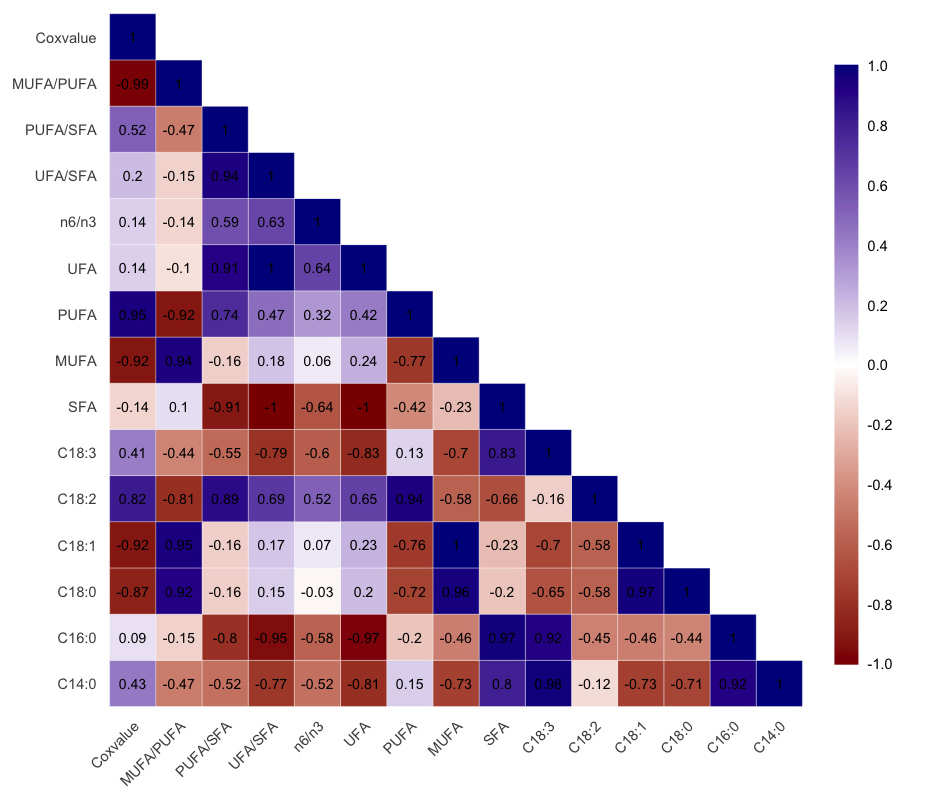


**Fig. S5.** Pearson correlation analysis of the identified fatty acid compounds from *G. tournefortii* L. seeds and their features at the beginning of seed production stage. C14:0 (myristic acid), C16:0 (palmitic acid), C18:0 (stearic acid), C18:1 (oleic acid), C18:2 (linoleic acid), C18:3 (linolenic acid), SFA (saturated fatty acids), MUFA (monounsaturated fatty acids), PUFA (polyunsaturated fatty acids), UFA (unsaturated fatty acids), n-6/n-3 (the ratio of linoleic acid to linolenic acid), UFA/SFA (the ratio of unsaturated fatty acids to saturated fatty acids), PUFA/SFA (the ratio of polyunsaturated fatty acids to saturated fatty acids), MUFA/PUFA (the ratio of monounsaturated fatty acid to polyunsaturated fatty acids), the cox value index


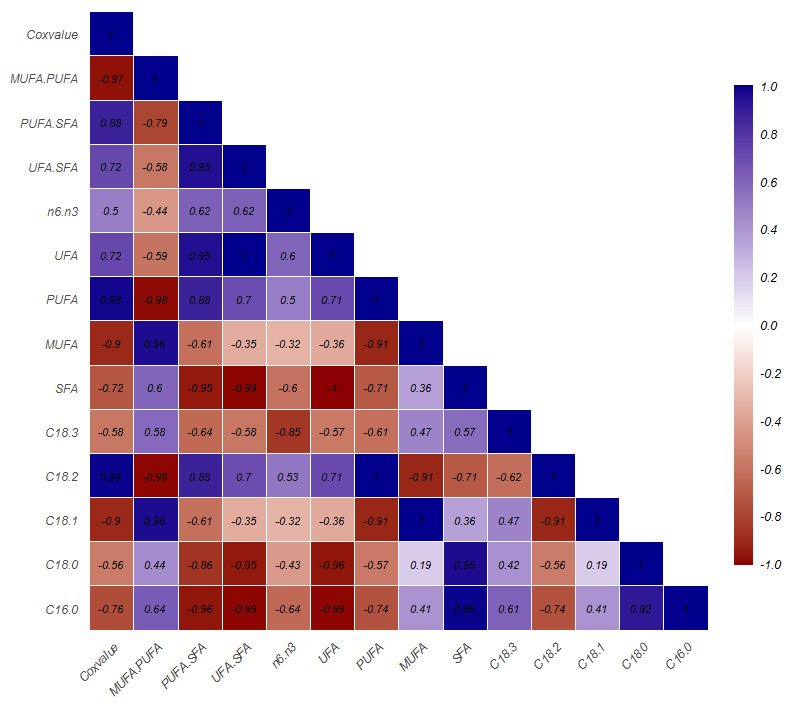


**Fig. S6.** Pearson correlation analysis of the identified fatty acid compounds from *G. tournefortii* L. seeds and their features at the end of seed production stage (List of the abbreviated factors used in this test has already memtioned in Fig. 5.).
